# Supplementary material for: Evidence for a postreproductive phase in female false killer whales Pseudorca crassidens
Source: Front Zool. 2017 Jun 21;14:30. doi: 10.1186/s12983-017-0208-y (PMC5479012; doi:10.1186/s12983-017-0208-y)
Supplement: Supplementary file 1 — Mean ovary weights (g per kg of estimated body mass) for non-pregnant, non-ovulating female false killer whales. Mean weight of both ovaries in grams per kilogram of estimated body mass as a function of age in a sample of non-pregnant, non-ovulating female false killer whales from Japan (n = 55). We found no evidence for a trend in mean ovary weight with age, though the power to detect any trends was low due to the small sample size. (PDF 57 kb) [file 12983_2017_208_MOESM1_ESM.pdf]

Mean ovary weights in female false killer whales.

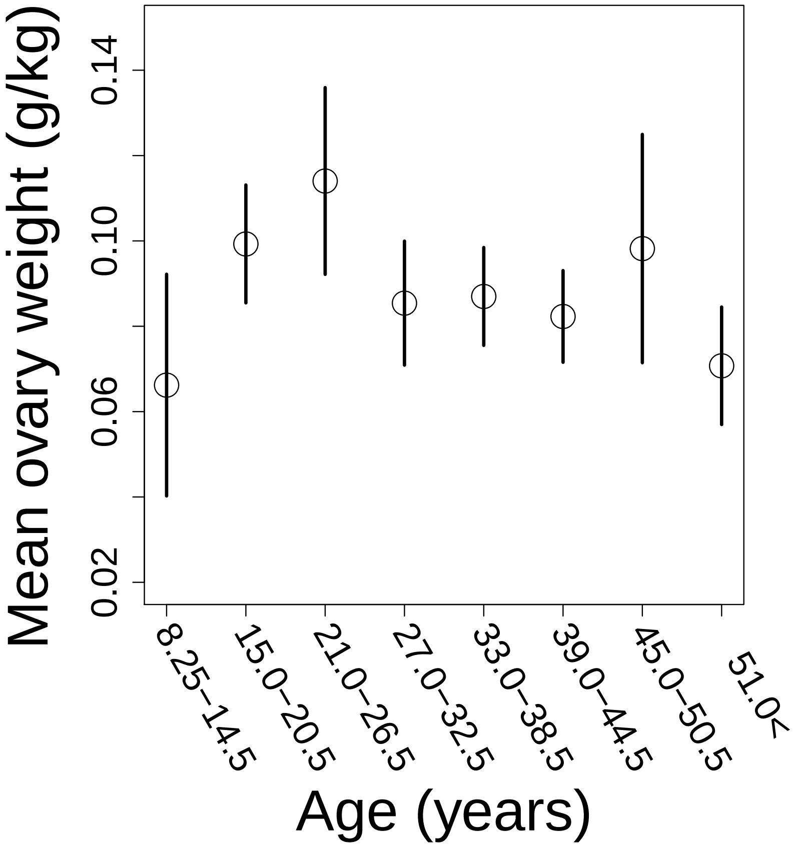

Additional file 1. Mean weight of both ovaries in grams per kilogram of estimated body mass as a function of age in a sample of non-pregnant, non-ovulating female false killer whales from Japan (n = 55). We found no evidence for a trend in mean ovary weight with age, though the power to detect any trends was low due to the small sample size.
