# Supplementary material for: Evidence for a postreproductive phase in female false killer whales Pseudorca crassidens
Source: Front Zool. 2017 Jun 21;14:30. doi: 10.1186/s12983-017-0208-y (PMC5479012; doi:10.1186/s12983-017-0208-y)
Supplement: Supplementary file 2 — Thickness of mammary gland in false killer whales in relation to age and reproductive status. Thickness of mammary gland in false killer whales from South Africa in relation to age and reproductive status. Open circles are used to represent animals that were not lactating (NL) and closed circles animals for those that were lactating (L). We fitted a linear regression model to mammary gland thickness with age and reproductive class (lactating, non-lactating) as explanatory variables. The dashed horizontal line is the fitted mean mammary gland thickness in non-lactating animals, and the solid line is the fitted mean thickness for lactating animals. The grey bands around each fitted mean are the 95% confidence intervals for the estimate. The figure shows that there is some evidence for greater mammary gland thickness in lactating animals but there was no evidence for a change with age in either. (PDF 47 kb) [file 12983_2017_208_MOESM2_ESM.pdf]

Thickness of mammary gland in false killer whales in relation to age and reproductive status.

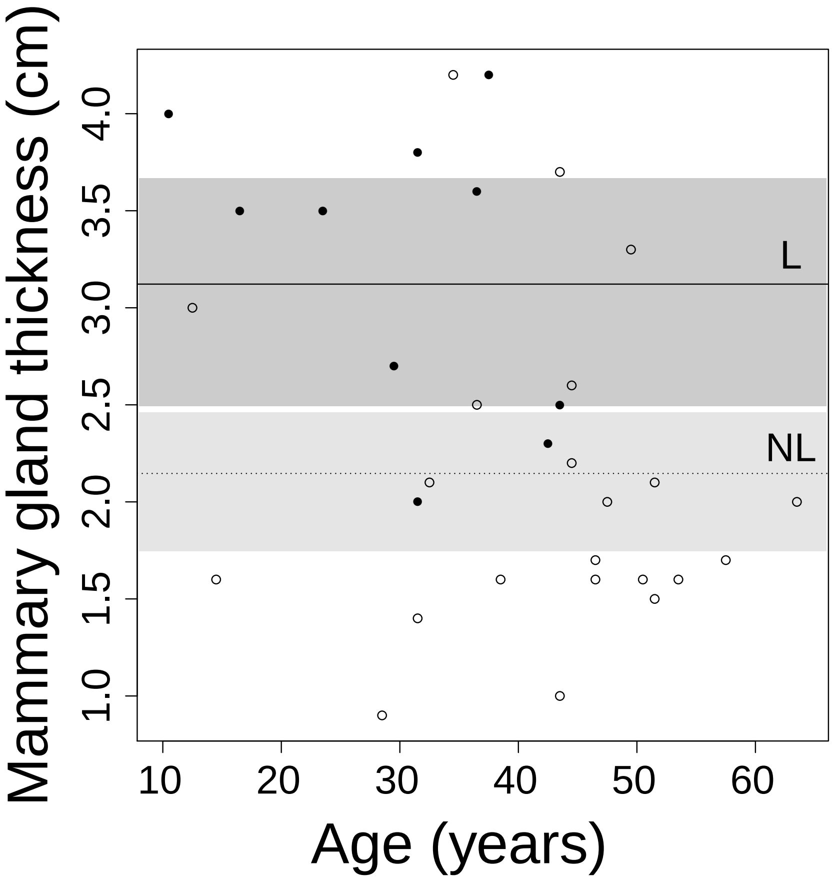

Additional file 2. Thickness of mammary gland in false killer whales from South Africa in relation to age and reproductive status. Open circles are used to represent animals that were not lactating (NL) and closed circles animals for those that were lactating (L). We fitted a linear regression model to mammary gland thickness with age and reproductive class (lactating, non-lactating) as explanatory variables. The dashed horizontal line is the fitted mean mammary gland thickness in non-lactating animals, and the solid line is the fitted mean thickness for lactating animals. The grey bands around each fitted mean are the 95% confidence intervals for the estimate. The figure shows that there is some evidence for greater mammary gland thickness in lactating animals but there was no evidence for a change with age in either.
