# Supplementary material for: Evidence for a postreproductive phase in female false killer whales Pseudorca crassidens
Source: Front Zool. 2017 Jun 21;14:30. doi: 10.1186/s12983-017-0208-y (PMC5479012; doi:10.1186/s12983-017-0208-y)
Supplement: Supplementary file 3 — The number of corpora lutea that represent pregnancies (CLP) and ovulation (CLO) as a function of age in false killer whales. There were only 13 individuals in each age group (total n = 26) from the combined dataset from Japan and South Africa so it is not possible to say anything conclusive about the trend in the corpora lutea of pregnancy and ovulation as function of age. (PDF 56 kb) [file 12983_2017_208_MOESM3_ESM.pdf]

The number of corpora lutea that represent pregnancies (CLP) and ovulation (CLO) as a function of age in false killer whales.

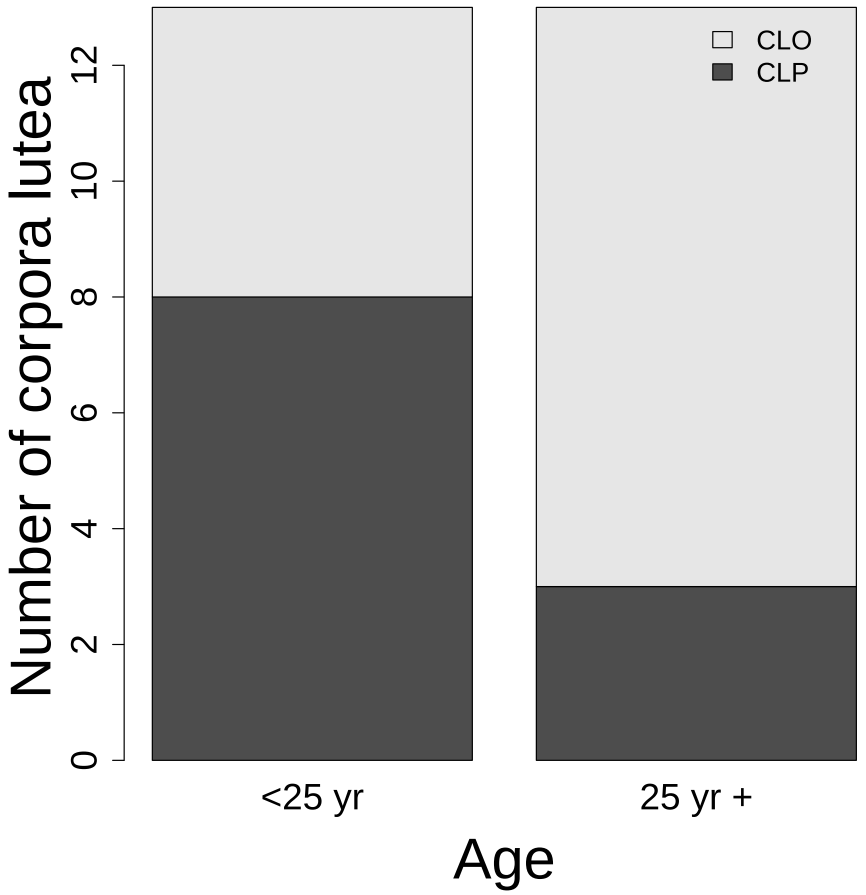

Additional file 3. There were only 13 individuals in each age group ($n = 26$) from the combined dataset from Japan and South Africa so it was not possible to say anything conclusive about the trend in the corpora lutea of pregnancy and ovulation as function of age.
