# Supplementary material for: Evidence for a postreproductive phase in female false killer whales Pseudorca crassidens
Source: Front Zool. 2017 Jun 21;14:30. doi: 10.1186/s12983-017-0208-y (PMC5479012; doi:10.1186/s12983-017-0208-y)
Supplement: Supplementary file 4 — Life table for Pseudorca crassidens. The life table for the combined dataset for specimens from Japan and South Africa with 1 year wide age classes, based on the modelled age frequency distribution. x is the upper limit of the age interval, e.g., 7-8 years of age appears as 8. f is the age frequency, the number of animals of each age class in the original data, F denotes the total number of animals at least x years of age, fp denotes the fitted values from the model for the number of animals in each age class, Fp denotes the total number of animals aged x or older, based on fp, l is the survivorship of animals of aged x, d is the frequency of mortality of animals aged x, px is the age-specific survival rate, q is the age-specific mortality rate, Lx is the number of individual-years lived between the ages of x and x+1, ex is the age-specific life expectancy, the number of years an individual aged x is expected to still live, Pr is the number of confirmed postreproductive animals. (PDF 72 kb) [file 12983_2017_208_MOESM4_ESM.pdf]

Life table for *Pseudorca crassidens*

Additional file 4. The life table for the combined dataset for specimens from Japan and South Africa with 1 year wide age classes, based on the modelled age frequency distribution. x is the upper limit of the age interval, e.g., 7-8 years of age appears as 8. f is the age frequency, the number of animals of each age class in the original data, F denotes the total number of animals at least x years of age, fp (for f') denotes the fitted values from the model for the number of animals in each age class, Fp (for F') denotes the total number of animals aged x or older, based on fp, l is the survivorship of animals of aged x, d is the frequency of mortality of animals aged x, p is the age-specific survival rate, q is the age-specific mortality rate, L is the number of individual-years lived between the ages of x and $x+1$, e is the age-specific life expectancy, the number of years an individual aged x is expected to still live, Pr is the number of confirmed postreproductive animals.

x	f	F	fp	Fp	l	d	p	q	L	e	Pr
8	1	91	1.94	91.00	1.00	0.02	0.98	0.02	1.93	47.03	0
9	1	90	1.92	89.06	0.98	0.02	0.98	0.02	1.92	46.33	0
10	3	87	1.91	87.14	0.96	0.02	0.98	0.02	1.90	45.63	0
11	0	87	1.90	85.23	0.94	0.02	0.98	0.02	1.89	44.92	0
12	1	86	1.89	83.33	0.92	0.02	0.98	0.02	1.88	44.21	0
13	0	86	1.87	81.45	0.90	0.02	0.98	0.02	1.87	43.49	0
14	3	83	1.86	79.58	0.87	0.02	0.98	0.02	1.85	42.77	0
15	1	82	1.85	77.72	0.85	0.02	0.98	0.02	1.84	42.05	0
16	3	79	1.84	75.87	0.83	0.02	0.98	0.02	1.83	41.32	0
17	3	76	1.82	74.03	0.81	0.02	0.98	0.02	1.82	40.58	0
18	2	74	1.81	72.21	0.79	0.02	0.97	0.03	1.81	39.84	0
19	2	72	1.80	70.40	0.77	0.02	0.97	0.03	1.79	39.10	0
20	1	71	1.79	68.59	0.75	0.02	0.97	0.03	1.78	38.35	0
21	3	68	1.78	66.81	0.73	0.02	0.97	0.03	1.77	37.59	0
22	1	67	1.77	65.03	0.71	0.02	0.97	0.03	1.76	36.83	0
23	1	66	1.75	63.26	0.70	0.02	0.97	0.03	1.75	36.07	0
24	4	62	1.74	61.51	0.68	0.02	0.97	0.03	1.74	35.30	0
25	0	62	1.73	59.77	0.66	0.02	0.97	0.03	1.73	34.53	0
26	1	61	1.72	58.04	0.64	0.02	0.97	0.03	1.71	33.75	0
27	0	61	1.71	56.32	0.62	0.02	0.97	0.03	1.70	32.96	0
28	2	59	1.70	54.61	0.60	0.02	0.97	0.03	1.69	32.17	0
29	1	58	1.69	52.91	0.58	0.02	0.97	0.03	1.68	31.38	0
30	4	54	1.68	51.22	0.56	0.02	0.97	0.03	1.67	30.58	0
31	3	51	1.66	49.55	0.54	0.02	0.97	0.03	1.66	29.77	0
32	2	49	1.65	47.88	0.53	0.02	0.97	0.03	1.65	28.96	0
33	0	49	1.64	46.23	0.51	0.02	0.96	0.04	1.64	28.15	0
34	2	47	1.63	44.59	0.49	0.02	0.96	0.04	1.63	27.32	1
35	1	46	1.62	42.96	0.47	0.02	0.96	0.04	1.62	26.50	0
36	3	43	1.61	41.34	0.45	0.02	0.96	0.04	1.61	25.66	1
37	4	39	1.60	39.73	0.44	0.02	0.96	0.04	1.59	24.83	0
38	4	35	1.59	38.13	0.42	0.02	0.96	0.04	1.58	23.98	1
39	1	34	1.58	36.54	0.40	0.02	0.96	0.04	1.57	23.13	0
40	1	33	1.57	34.96	0.38	0.02	0.96	0.04	1.56	22.28	0
41	1	32	1.56	33.39	0.37	0.02	0.95	0.05	1.55	21.42	0
42	1	31	1.55	31.83	0.35	0.02	0.95	0.05	1.54	20.55	0
43	5	26	1.54	30.28	0.33	0.02	0.95	0.05	1.53	19.68	0
...	...	...	...	...	...	...	...	...	...	...	...

(Additional file 4 continued)

x	f	F	fp	Fp	l	d	p	q	L	e	Pr
...	...	...	...	...	...	...	...	...	...	...	...
44	3	23	1.53	28.74	0.32	0.02	0.95	0.05	1.52	18.81	0
45	0	23	1.52	27.21	0.30	0.02	0.94	0.06	1.51	17.92	0
46	2	21	1.51	25.69	0.28	0.02	0.94	0.06	1.50	17.03	2
47	2	19	1.50	24.19	0.27	0.02	0.94	0.06	1.49	16.14	0
48	1	18	1.49	22.69	0.25	0.02	0.93	0.07	1.48	15.24	0
49	1	17	1.48	21.20	0.23	0.02	0.93	0.07	1.47	14.33	0
50	2	15	1.47	19.72	0.22	0.02	0.93	0.07	1.46	13.42	1
51	2	13	1.46	18.25	0.20	0.02	0.92	0.08	1.46	12.50	0
52	1	12	1.45	16.79	0.18	0.02	0.91	0.09	1.45	11.58	0
53	3	9	1.44	15.34	0.17	0.02	0.91	0.09	1.44	10.65	1
54	1	8	1.43	13.90	0.15	0.02	0.90	0.10	1.43	9.71	1
55	2	6	1.42	12.47	0.14	0.02	0.89	0.11	1.42	8.77	1
56	2	4	1.41	11.05	0.12	0.02	0.87	0.13	1.41	7.82	2
57	1	3	1.40	9.63	0.11	0.02	0.85	0.15	1.40	6.86	1
58	0	3	1.39	8.23	0.09	0.02	0.83	0.17	1.39	5.90	0
59	0	3	1.39	6.84	0.08	0.02	0.80	0.20	1.38	4.94	0
60	0	3	1.38	5.45	0.06	0.02	0.75	0.25	1.37	3.96	0
61	0	3	1.37	4.07	0.04	0.02	0.66	0.34	1.36	2.98	0
62	1	2	1.36	2.71	0.03	0.01	0.50	0.50	1.35	1.99	1
63	1	1	1.35	1.35	0.01	0.01			0.67	1.00	1
