# Supplementary material for: Evidence for a postreproductive phase in female false killer whales Pseudorca crassidens
Source: Front Zool. 2017 Jun 21;14:30. doi: 10.1186/s12983-017-0208-y (PMC5479012; doi:10.1186/s12983-017-0208-y)
Supplement: Supplementary file 5 — Fitted data from smooth regression models for age-specific fecundity. Age-specific fecundity data had to be smoothed to generate values for each single-year age class in the survival dataset to generate the life table and carry out further analyses. It was not clear what value should be used for the degrees of freedom, in other words how smooth the plots should be, so we generated curves under 10 different scenarios for the degrees of freedom; a: df=2.1, b: df=2.9, c: df=3.7, d: df=4.5, e: df=5.3, f: df=6.1, g: df=6.9, h: df=7.7, i: df=8.5, j: df=8.9. The plot on the left shows the curves for the combined dataset and the plots on the right show the curves for the separate datasets. (PDF 54 kb) [file 12983_2017_208_MOESM5_ESM.pdf]

Fitted data from smooth regression models for age-specific fecundity.

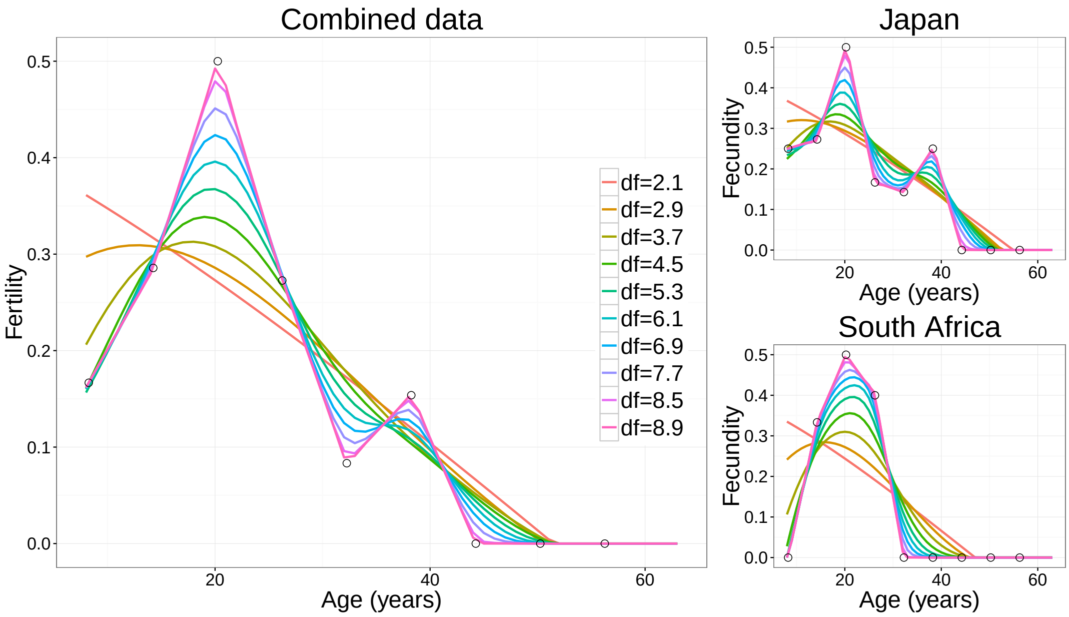

Additional file 5. Age-specific fecundity data had to be smoothed to generate values for each single-year age class in the survival dataset to generate the life table and carry out further analyses. It was not clear what value should be used for the degrees of freedom, in order words how smooth the plots should be, so we generated curves under ten different scenarios for the degrees of freedom; a: df=2.1, b: df=2.9, c: df=3.7, d: df=4.5, e: df=5.3, f: df=6.1, g: df=6.9, h: df=7.7, i: df=8.5, j: df=8.9. The plot on the left shows the curves for the combined dataset and the plots on the right show the curves for the separate datasets.
