# Supplementary material for: Evidence for a postreproductive phase in female false killer whales Pseudorca crassidens
Source: Front Zool. 2017 Jun 21;14:30. doi: 10.1186/s12983-017-0208-y (PMC5479012; doi:10.1186/s12983-017-0208-y)
Supplement: Supplementary file 6 — Dataset of fitted values of age-specific fecundity under 10 different smoothing scenarios for the fecundity data from Japan. (ZIP 64 kb) [file 12983_2017_208_MOESM6_ESM.zip › Additional files_Annotated_6.pdf]

Datasets of fitted values of age-specific fecundity.

Additional file 6. Dataset of fitted values of age-specific fecundity under ten different smoothing scenarios for the fecundity data from Japan:

`AF6_Japan_fecundity_models.txt`

Additional file 7. Dataset of fitted values of age-specific fecundity under ten different smoothing scenarios for the fecundity data from South Africa:

`AF7_SouthAfrica_fecundity_models.txt`

Additional file 8. Dataset of fitted values of age-specific fecundity under ten different smoothing scenarios for the fecundity data from the combined data from the two populations:

`AF8_Combinedpopulation_fecundity_models.txt`

These data were used to construct the plots in Additional File 5.
