# Supplementary material for: Evidence for a postreproductive phase in female false killer whales Pseudorca crassidens
Source: Front Zool. 2017 Jun 21;14:30. doi: 10.1186/s12983-017-0208-y (PMC5479012; doi:10.1186/s12983-017-0208-y)
Supplement: Supplementary file 7 — Dataset of fitted values of age-specific fecundity under 10 different smoothing scenarios for the fecundity data from South Africa. (PDF 63 kb) [file 12983_2017_208_MOESM7_ESM.pdf]

The estimated PrR and the distribution of PrR under the null hypothesis of no PRLS for false killer whales from Japan and South Africa

Additional file 9. The distribution of postreproductive representation, PrR, under the null hypothesis of no postreproductive life span, PRLS, is shown with a black line in each plot, with the 99% confidence interval shaded grey, and the estimated PrR marked with a red dashed line. Each plot represents the calculation of PrR using smoothed fecundity with ten different values for the degrees of freedom in the model (i.e., the smoothness); a: df=2.1, b: df=2.9, c: df=3.7, d: df=4.5, e: df=5.3, f: df=6.1, g: df=6.9, h: df=7.7, i: df=8.5, j: df=8.9.

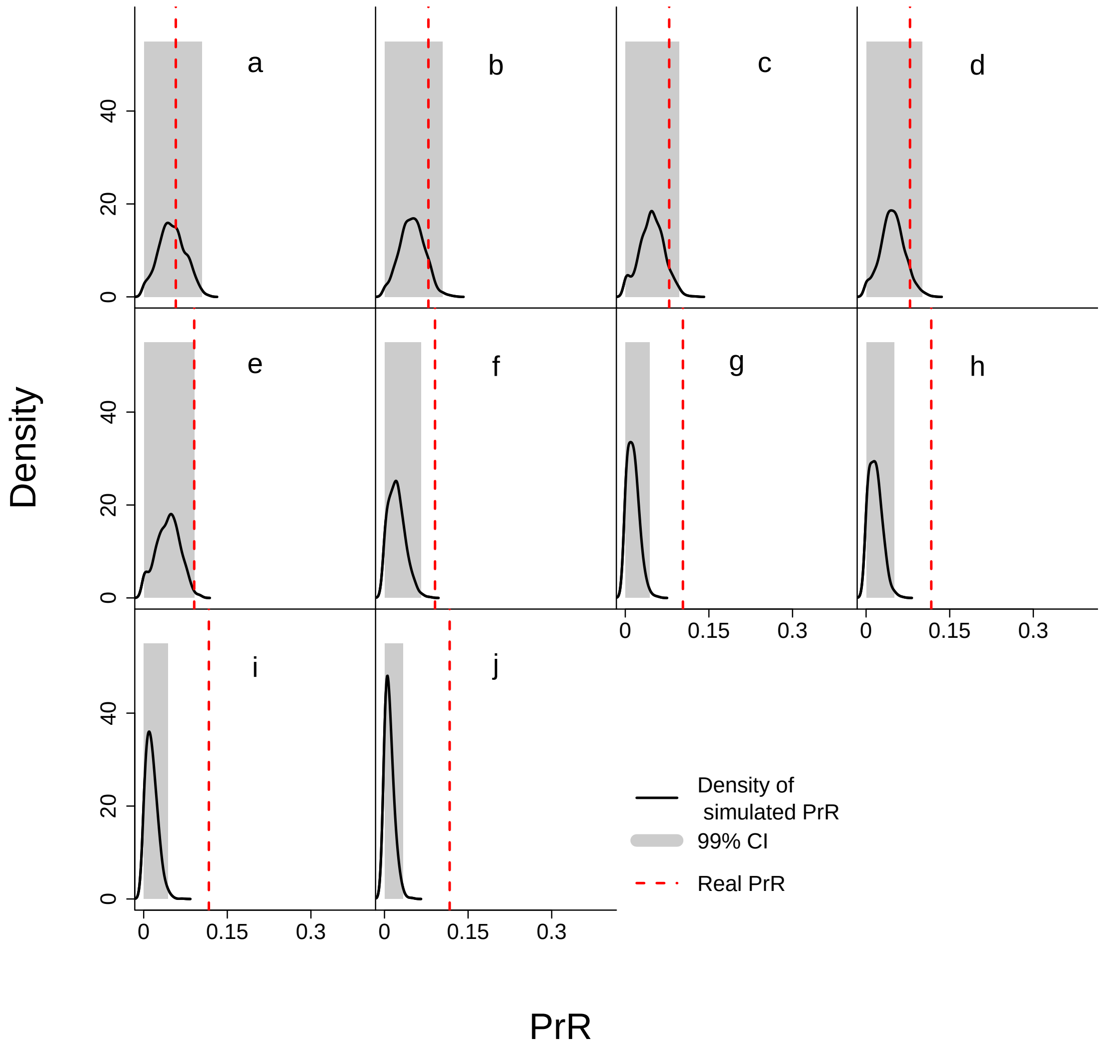

Additional file 9a. Japan

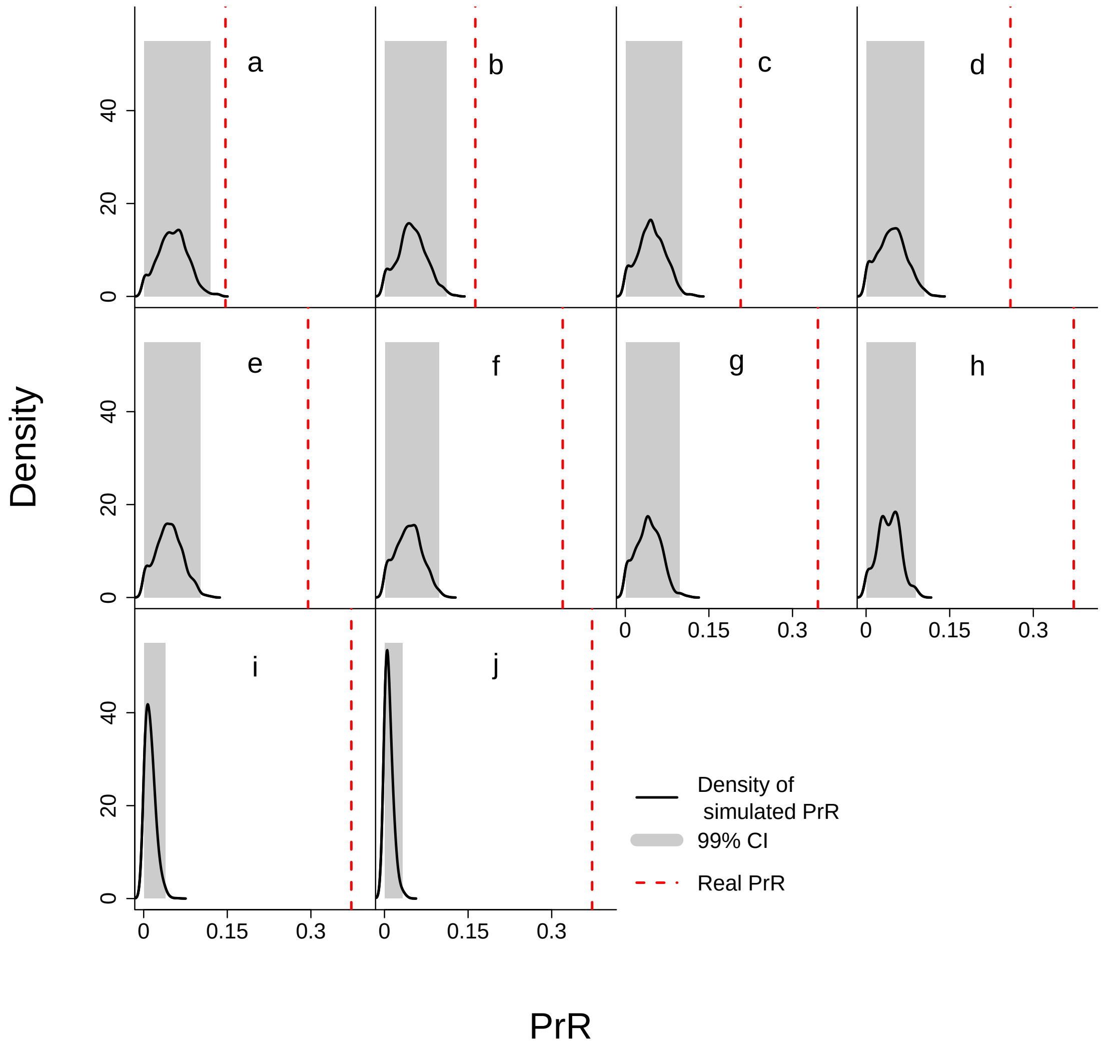

Additional file 9b. South Africa
